# Feasibility of a co-designed and personalised intervention to improve vegetable intake in rural-dwelling young adults

**DOI:** 10.1186/s12966-025-01796-7

**Published:** 2025-07-14

**Authors:** Katherine Mary Livingstone, Jonathan C. Rawstorn, Stephanie R. Partridge, Yuxin Zhang, Eric O, Stephanie L. Godrich, Sarah A. McNaughton, Gilly A. Hendrie, Kathleen M. Dullaghan, Gavin Abbott, Lauren C. Blekkenhorst, Ralph Maddison, Scott Barnett, John C. Mathers, Laura Alston

**Affiliations:** 1 Institute for Physical Activity and Nutrition (IPAN), School of Exercise and Nutrition Sciences, Deakin University, Geelong, VIC 3220 Australia; 2 Susan Wakil School of Nursing and Midwifery, Faculty of Medicine and Health, The University of Sydney, NSW 2006 Sydney, Australia; 3 Charles Perkins Centre, The University of Sydney, Sydney, NSW Australia; 4 Digital Services, DS Digital Engagement, Deakin University, Geelong, VIC 3220 Australia; 5 Nutrition and Health Innovation Research Institute, School of Medical and Health Sciences, Edith Cowan University, Bunbury, WA 6230 Australia; 6 Health and Well-Being Centre for Research Innovation, School of Human Movement and Nutrition Sciences, University of Queensland, St Lucia, QLD 4067 Australia; 7 School of Exercise and Nutrition Sciences, Deakin University, Geelong, VIC 3220 Australia; 8 Human Health Program, Health & Biosecurity, CSIRO, Adelaide, SA 5000 Australia; 9 Nutrition and Health Innovation Research Institute, School of Medical and Health Sciences, Edith Cowan University, WA 6027 Perth, Australia; 10 Medical School, Royal Perth Hospital Unit, The University of Western Australia, WA 6000 Perth, Australia; 11 Applied Artificial Intelligence Institute (A2I2), Deakin University, VIC 3220 Geelong, Australia; 12 Human Nutrition & Exercise Research Centre, Centre for Healthier Lives, Population Health Sciences Institute, Newcastle University, Newcastle upon Tyne, NE2 4HH UK; 13 Deakin Rural Health, School of Medicine, Faculty of Health, Deakin University, VIC 3280 Warrnambool, Australia; 14 Research Unit, Colac Area Health, Colac, VIC 3251 Australia

**Keywords:** Rural, Young adults, Vegetable intake, Diet, Intervention

## Abstract

**Background:**

This study determined the feasibility, acceptability, engagement and efficacy of a co-designed and personalised digital intervention to increase vegetable intake (*Veg4Me*) in young (18-to-35 years) rural-dwelling Australian adults.

**Methods:**

Participants living in rural Australia were recruited via local government networks and social media and randomised to receive 12-weeks’ access to personalised (intervention) or non-personalised (control) versions of the free *Veg4Me* web application. The intervention included: (1) personalised recipes, (2) geo-located food environment map, (3) healthy eating resources, (4) goal-setting portal, and (5) personalised e-newsletters. The primary outcome was feasibility (recruitment, participation, and retention rate). Secondary outcomes were user engagement, acceptability, and changes in dietary intake and habits. Descriptive statistics were presented for the intervention and control groups. Generalised linear models estimated group differences in outcomes at 12-weeks.

**Results:**

Of the 125 eligible individuals who registered *Veg4Me* accounts, 116 were randomised and 83 completed postintervention data collection. Recruitment, participation and retention rates were 47%, 93% and 72%, respectively. Intervention participants had higher engagement (median 20 [IQR 3, 54] vs. 6 [IQR 1, 28] page visits/week) and acceptability of the intervention (76%; vs. 52%) than control. Almost all intervention participants liked having access to the recipe library (93%) and reported that the e-newsletters prompted them to access the intervention (90%). Most accessed the goal-setting function (78%), food environment map (76%), and healthy eating resources (63%). More intervention participants reported their vegetable intake had changed in the last 12 weeks, compared with the control (85% vs. 57%; *p* = 0.010). Mean vegetable intake at 12 weeks for intervention and control was 2.73 (SD 1.1) and 2.66 (SD 1.4) serves/day, respectively (*p* = 0.76). At 12 weeks, for the intervention and control, confidence to shop regularly for nutritious foods was 68% and 55% (*p* = 0.09), to cook root vegetables was 88% and 81% (*p* = 0.11), and to cook pulses was 54% and 48%, respectively (*p* = 0.52).

**Conclusions:**

A co-designed and personalised digital intervention to increase vegetable intake was feasible, engaging and acceptable among rural-dwelling young adults. Although change in reported vegetable intake was small, findings showed promise for improving dietary intake and habits. Larger trials of effectiveness are needed to determine whether personalised digital interventions can help address health inequities experienced by rural-dwelling young adults.

**Trial registration:**

Australia New Zealand Clinical Trials Registry, ACTRN12623000179639, prospectively registered on 21/02/2023, according to the World Health Organizational Trial Registration Data Set. Universal Trial Number U1111-1284-9027.

**Supplementary Information:**

The online version contains supplementary material available at 10.1186/s12966-025-01796-7.

## Introduction

Health inequities are more persistent in rural areas compared with urban areas, including up to 40% higher rates of chronic disease in rural communities in high income countries such as Australia [1, 2]. Rural communities experience inequities in accessing healthy food. This leads to additional challenges in obtaining a healthy diet when compared with their urban counterparts, which in turn exacerbates health inequities [3, 4]. As a result, less than 11% of rural-dwelling adults in Australia consume the recommended 5–6 serves of vegetables daily, and vegetable intake is lowest in young adults aged 18–35 years [5]. Targeting an increase in consumption of healthy foods, such as vegetables, has been shown to be a more effective behaviour change strategy in young adults than targeting a decrease in unhealthy foods, while also improving overall diet quality [6, 7]. Interventions to increase vegetable intake frequently target fruit intake concurrently yet typically result in increased fruit rather than vegetable intake [8]. Focussing on the distinct barriers to vegetable intake represents an opportunity to prevent further widening of health disparities for the current and future generations of rural Australians [9]. 

Digital intervention delivery is an accessible and scalable strategy that has potential to increase vegetable intake in rural communities [10, 11]. However, to be effective, digital interventions must align with the users’ digital literacy, self-efficacy, technology access, and attitudes towards use, i.e., digital health equity [12]. Personalising digital interventions to user needs may help improve digital health equity [13], and may achieve greater changes in dietary intake than generalised dietary interventions [14]. However, long-term engagement is a commonly reported challenge [15], which may be attributable to a lack of consumer engagement during the design of the intervention [16]. 

There is limited data on effective digital interventions to improve vegetable intake in rural communities [17], and no interventions have been specifically designed to meet the needs of young adults living in rural areas [16]. To address this, we co-designed the *Veg4Me* web application (app) [18], with young adults aged 18–35 years, rural local governments, the National Heart Foundation of Australia, web developers and the research team. *Veg4Me* aims to address individual and food environment barriers to vegetable intake by providing access to personalised food literacy resources and behaviour change support as well as information on the user’s local food environment. The primary aim of this study was to determine the feasibility of delivering *Veg4Me*. Secondary aims were to examine user engagement and acceptability, digital equity, and efficacy on vegetable intake and dietary habits.

## Materials and methods

### Study design

We conducted a prospectively registered (ACTRN12623000179639) 12-week assessor-blinded, two-arm, parallel randomised controlled trial from August 2023 until April 2024, consistent with the Consolidated Standards of Reporting Trials guidelines [19], and the Declaration of Helsinki. All participants enrolled in the study provided informed e-consent through the web app. The study protocol was published prior to completion of data collection [20]. Study reporting is guided by the Template for Intervention Description and Replication (TIDieR) checklist [21] and the Consolidated Standards of Reporting Trials (CONSORT) 2010 statement for feasibility trials [19] (Additional file 1).

### Study population

Eligible participants were aged 18–35 years (inclusive), living in the Loddon Campaspe region or Colac Otway Shire in Victoria, Australia (Modified Monash Model MM2 [regional centre] to MM5 [small rural town]) [22], currently consuming < 5 serves of vegetables/day, willing/able to access to an internet-connected mobile device or computer and the free *Veg4Me* web app, and able to provide informed e-consent to participate. The term ‘rural’ is used throughout to refer to these regions. Participants were excluded if English was not the main language spoken at home, they were pregnant or breastfeeding, or were currently participating in another research trial involving dietary and/or physical activity interventions. Pregnant or breastfeeding individuals were excluded based on the premise that women are likely to change their diet during this life stage due to factors such as food safety concerns, including preprepared salads/vegetables, food cravings/aversions, and dietary advice from health professionals. Participants with medical conditions that required specific clinical nutrition advice, such as type 1 diabetes, were required to obtain medical clearance from their general practitioner before participating.

### Recruitment, screening and baseline assessment

Potential participants were recruited via local government networks including distributing flyers with quick response (QR) codes in community and neighbourhood houses, libraries and sporting facilities, and online promotion in local government newsletters and social media channels. Paid advertisements on Facebook, Instagram and Twitter, targeted based on age, gender and location were used, as well as public Facebook groups and media releases via online and print articles in local and national media. Study advertisements included a link to the *Veg4Me* landing page (https://veg4me.deakin.edu.au), where interested individuals could directly access study information, including a Plain Language Statement and research team contact details. Individuals completed online screening questions during user account registration. A two-factor authentication process was used on the *Veg4Me* landing page requiring interested individuals to provide their email address then enter an emailed code before progressing to the Qualtrics survey platform to complete screening and, if eligible, baseline survey completion (demographic characteristics, dietary habits, vegetable intake, digital device use) and randomisation. If participants did not complete this two-factor authentication process then they were classified as lost to follow-up. Participants completing the 12-week intervention period (including follow-up survey) received a AUD75 eGift card (e.g., supermarket or general retail voucher). Participants who also completed the process evaluation interviews received an additional AUD20 eGift card.

### Randomisation and blinding

Participants were randomised in a 1:1 ratio to either the personalised (intervention group) or non-personalised (control group) version of *Veg4Me* using a computerised process. Randomisation occurred automatically via *Veg4Me*’s Qualtrics integration, in random permuted blocks (size 2 and 4), and stratified for region (Loddon Campaspe and Colac Otway Shire) and gender (male, female, other). The lead researcher (KML) and statistician (GA) performing the data analyses, and co-investigators, were blinded to randomisation code throughout the study. The project manager (KMD) had access to the randomisation code. Analyses for this manuscript were undertaken only when all data were collected.

### Intervention

Participants randomised to the intervention group received 12 weeks’ access to (1) personalised recipes, (2) a geo-located local food environment map, and (3) a healthy eating hub via the *Veg4Me* web app, as well as (4) behaviour change support using a Qualtrics goal-setting portal and e-newsletters delivered via email.

#### Personalised recipes

Each week, seven new personalised recipes were made available to participants that were centred around a featured vegetable of the week. As detailed elsewhere [20], recipes were personalised for dietary and cooking preferences collected in the baseline survey. Participants also had access to a browsable library of > 240 recipes provided with agreement by the National Heart Foundation of Australia.

#### Geo-located food environment map

A custom Google map with colour-coded icons identified local rural food resources within each local government area, including foodbanks, community gardens, cooking classes, community cafes and lunches, as well as markets identified by young adults, local governments and the research team during co-design workshops [18]. Participants could view any region, but the default location was set to their residential postcode collected in the baseline survey. The map included information on each resource, including contact details and opening hours [18]. The design was informed by the Capability, Opportunity and Motivation (COM-B) model of behaviour change [23], where access to physical resources helps facilitate healthy eating and social connection (i.e., opportunity to access healthy food options that are available locally).

#### Healthy eating resources

A healthy eating hub supported participants to develop knowledge, confidence, and skills (i.e. food literacy) to increase vegetable intake, by providing information on how to grow, choose, store and prepare vegetables. Aligned with the Capability, Opportunity and Motivation (COM-B) model of behaviour change [23], the provision of healthy eating resources (i.e. opportunity) was intended to increase self-efficacy to eat healthily (i.e. capability and motivation). These resources were not personalised but were available to the intervention group only.

#### Goal-setting portal

A goal-setting portal integrated via Qualtrics encouraged participants to set weekly SMART (Specific, Measurable, Achievable, Relevant, and Time-Bound) goals using a structured (drop-down options) or unstructured (free text box) approach. Research staff monitored unstructured goal setting relevance and provided ad hoc email support to ensure goals were relevant to the intervention. As detailed elsewhere [20], the > 20 structured goal types, that emerged from co-design workshops, included recipe goals (e.g., “Prepare at least two of the recipes from this week’s selection”), healthy eating goals (e.g., “Eat an additional serve of vegetables on every day of the week”), and food environment map goals (e.g., “Visit at least one of the places on my food environment map this week”). The goal-setting portal was informed by the Self-Determination Theory that focuses on promoting internal sources of motivation, personal growth and fulfillment [24], and the Goal Contents Theory to support autonomous healthy eating motivation.

#### e-Newsletters

Participants received a personalised weekly e-newsletter developed and disseminated by the research team. The purpose of the e-newsletters was to prompt engagement with *Veg4Me* resources (e.g., to access the healthy eating hub) and to support retention. Each e-newsletter included healthy eating tips, information about the vegetable of the week, with links to matched recipes and was personalised to the participant by including their name and a reminder of their weekly goal.

### Control

The control group received 12 weeks’ access to a non-personalised and limited version of *Veg4Me* which included the browsable recipe library (> 240 recipes) and a single list of community food resources for all rural areas but lacked the personalisation and access to the healthy eating resources, goal-setting portal and e-newsletters.

### Demographic characteristics

Demographic characteristics (birth date, gender, postcode, education, living arrangement, smoking status) were collected via an online survey at baseline and updated at 12-week follow-up as required. Postcode was used to derive rurality (regional; large rural town; small rural town) using the Modified Monash Model [22]. 

### Outcome measures

#### Primary (feasibility) outcome measures

Participant recruitment, participation and retention rates were used to examine the feasibility of delivering the intervention. Recruitment rate (%) was defined as the total number of individuals randomised/ total number who registered on the *Veg4Me* website after removal of responses deemed as fraudulent, and duplicate responses. Participation rate (%) was the total number of eligible participants randomised/ total number eligible and provided consent at baseline. Retention rate (%) was the total number of participants who completed postintervention data collection/ total number who were randomised into the study. Based on previous nutrition and health interventions in young adults and criteria for feasibility studies [25–27], we considered success as a minimum 40% recruitment rate, 70% participation rate and 80% retention rate.

#### Secondary outcome measures

As detailed elsewhere [20], secondary outcomes included engagement with and acceptability of *Veg4Me*, vegetable intake and dietary habits, as well as digital health equity (Table 1). Briefly, participants’ engagement with *Veg4Me* was collected continuously in both groups using the web app’s in-built tracking to record the number and duration of visits to all *Veg4Me* pages including the recipes, food environment resources, and healthy eating hub. Qualtrics data were used to track the use of the weekly goal-setting feature (number of participants setting goals and goal type). The 16-item Post-Study System Usability Questionnaire (PSSUQ) [28] was administered via Qualtrics at follow-up to measure acceptability of *Veg4Me*. Responses were collected using 7-point Likert scale ranging from “strongly agree” to “strongly disagree”. Questions specifically designed for use in this study were used to understand experience with the recipes, food environment resources, healthy eating resources, newsletters, goal-setting function and *Veg4Me* overall [20]. Data on acceptability were collected using 5-point Likert scale responses from “strongly agree” to “strongly disagree” to determine whether participants agreed with the statements on recipes (e.g. the recipes were easy to access) and whether participants agreed that the food environment and healthy eating hub resources were useful (e.g. the community garden resource was useful). Acceptability of the goal-setting function and e-newsletters was assessed based on whether participants reported using the goal-setting function (yes/no) and that the e-newsletters prompted them to access the *Veg4Me* web application (yes/no/I didn’t read any e-newsletters).

Information on efficacy outcomes (vegetable intake and dietary habits) were collected via an online survey (Qualtrics) at baseline and at follow-up. Estimated usual serves of vegetables consumed per day were assessed with a brief diet questionnaire that was validated against weighed food records [29]. Information was collected on self-reported cooking confidence [30], self-efficacy to eat healthy foods [31] and self-efficacy to eat vegetables [32] using 5-point Likert scales. Participants were asked to identify their readiness for change in eating habits according to the five stages of the transtheoretical model of change, i.e. pre-contemplation, contemplation, preparation, action and maintenance [33]. Information on self-reported changes in usual eating habits, food preparation habits and shopping habits over the past 12-weeks was requested at follow-up, as well information on what participants needed to help them increase their vegetable intake, which had been previously adapted from the COM-B model [34]. Details of response options are provided elsewhere [20]. 

Information on digital health equity was collected via Qualtrics at baseline and at follow-up [12]. Questions captured digital health equity metrics at the individual level, spanning digital literacy, digital self-efficacy, technology access and attitudes towards technology use.

#### Process evaluation

Post intervention, semi-structured 20–30-minute interviews were conducted via Zoom with participants who consented in the baseline survey to be contacted for this purpose. Interview participants were sampled from both the intervention and control groups to gain a holistic understanding of participants’ experiences, although participants in the intervention group were prioritised to gain insights into intervention features. Participants were sampled on a rolling basis from across the strata groups (region and gender). Based on Braun and Clarke’s recommendations [35], 20 participants were deemed sufficient unless saturation was reached. Interviewer questions focused on overall impressions of *Veg4Me*, as well as specific components of the intervention, such as the recipes and food environment resources. Data from the 12-week PSSUQ were used to explore items that received high and low usability scores. Participants were also asked to describe any barriers or enablers in using *Veg4Me*.

### Sample size

The recruitment target was 150 participants (75 per group) based on an anticipated dropout rate of 20%, and it was expected that 120 participants (60 per group) would complete the trial [25–27, 36, 37]. This pragmatic sample size was based on good practice guidelines for feasibility trials [38]. 

### Data analysis

Data were stored on password-protected Deakin University electronic servers and de-identified by assigning a unique identification number to each participant. Data analyses was conducted on an intention to treat basis in Stata (Version SE 17.0; StataCorp). Feasibility outcomes were compared with benchmarks of success (see Primary [feasibility] outcome measures). Fraudulent responses were identified by the research team based on a combination of the following: multiple responses in short period of time, short survey duration; IP addresses / locations duplications; formulaic email addresses; atypical names of respondents; Qualtrics-identified spam/duplicate responses; illogical responses. Mean and SD were calculated for continuous variables and n and % for categorical variables. Generalised linear and logistic models were used to evaluate group differences on outcomes at 12 weeks adjusted for baseline levels of the outcome (where available), age and stratifying factors (gender and region). Likert scales were converted to binary variables (agree vs. neutral/ disagree; confident vs. neutral/ not confident) for use in these models. In the primary analysis, missing outcome data were handled using multiple imputation by chained equations with stratification factors and age included as auxiliary variables, with imputation performed separately by study group [39]. The stratification factor region was collinear (perfect prediction) with the confidence to cook potatoes outcome and so was omitted as a covariate in estimation and imputation models for this outcome. Similarly, the stratification factors gender and region were collinear with the confidence to cook fresh vegetables and so were omitted. Sensitivity analyses included a complete case analysis of 12-week intervention effects and estimation of intervention effects stratified by high and low engagement (≥ 10 vs. < 10 median pages visits/week) and by rurality (regional vs. rural).

Interviews were recorded, transcribed verbatim and any identifying information removed. The researcher who developed the questionnaire items (KML) read the responses to identify themes in the data and undertook inductive content analysis. The identified themes were then confirmed by a second researcher (KMD). Example quotes from participants were selected to reflect acceptability of intervention features. The reporting of the qualitative data was guided by the Consolidated criteria for reporting qualitative research (COREQ) checklist [40]. Any adverse events were recorded and reported to the Chair of the Deakin University Ethics Committee.

## Results

### Feasibility

#### Participant recruitment, participation and retention

**Fig. 1:**
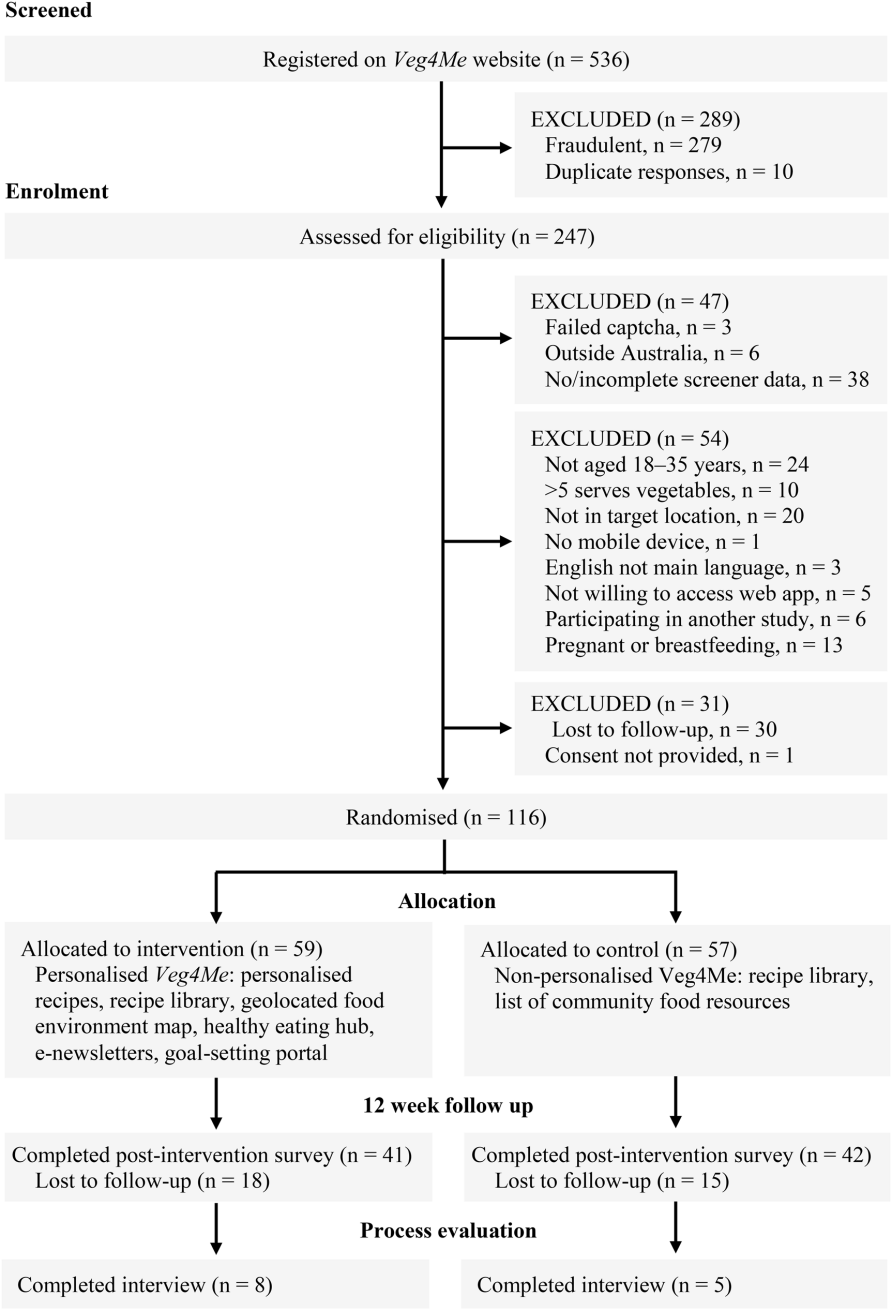
CONSORT diagram describing study design and flow of participants through the 12-week *Veg4Me* randomised controlled trial

Figure 1 illustrates the study design and flow of participants through the 12-week *Veg4Me* RCT. A total of *n* = 536 accounts were registered on the *Veg4Me* website. After removal of those classified as fraudulent and/or duplicate responses (*n* = 289), *n* = 125 were eligible and provided consent to participate, *n* = 116 were randomised and *n* = 83 completed postintervention data collection. A total of *n* = 13 participants (*n* = 8 intervention; *n* = 5 control) completed process evaluation interviews, which was less than the *n* = 20 target estimated due to achieving data saturation. Recruitment commenced in August 2023 and ceased February 2024. Recruitment ceased before the targeted sample size of 150 participants was reached due to funding requirements. Data collection ceased in May 2024, 12 weeks after the final participant was recruited. No adverse events were reported by participants.

The recruitment and participation rates were 47% (116 randomised/ 247 legitimate registered accounts) and 93%, respectively (116 randomised/ 125 eligible). The overall retention rate at 12 weeks was 72% (*n* = 83), with 70% (*n *=  41) in the intervention group and 74% (*n* = 42) in the control group.

### Participant characteristics

The baseline characteristics of the randomised participants are presented in Table 1. The majority were female (85%) and born in Australia (88%). Approximately one third of participants resided in regional centres (39%), one third in large rural towns (34%) and one third in small rural towns (28%). A total of 57% lived with a partner, 40% had a high level of education, 30% self-reported excellent or very good health and 10% were current smokers. Mean (SD) vegetable intake was 2.54 (1.20) serves/day. Most participants did the main food shopping (77%) and food preparation (71%) for the household. The mean number of meals eaten with household members in the last 7 days was 8.94 (5.06). The largest number of participants (35%) were at the ‘contemplation’ stage of behaviour change, i.e. seriously intending to improve eating habits in the next 6 months, while 29% were at the ‘action’ stage, i.e. doing something to improve eating habits.

**Table 1:** Characteristics of participants randomised into *Veg4Me* at baseline

Characteristic	Total (*n* = 116)	Control (*n* = 57)	Intervention (*n* = 59)
**Socio-demographics and health**			
Age, years, mean (SD)	28.1 (4.90)	28.5 (4.67)	27.6 (5.12)
Female, n (%)	98 (84.5)	48 (84.2)	50 (84.8)
Born in Australia, n (%)	102 (87.9)	50 (87.7)	52 (88.1)
Rurality, n (%)^1^			
Regional	45 (38.8)	22 (38.6)	23 (39.0)
Large rural towns	39 (33.6)	20 (35.1)	19 (32.2)
Small rural towns	32 (27.6)	15 (26.3)	17 (28.8)
Education, n (%)^2^			
High	46 (39.7)	13 (22.8)	23 (39.0)
Medium	34 (29.3)	12 (36.8)	13 (22.0)
Low	36 (31.0)	23 (40.4)	23 (39.0)
Self-reported health			
Excellent / Very good	35 (30.2)	17 (29.8)	18 (30.5)
Good	57 (49.1)	32 (56.1)	25 (42.4)
Fair / Poor	24 (20.7)	8 (14.0)	16 (27.1)
Smoking status			
Current smoker	12 (10.3)	4 (7.02)	8 (13.6)
Used to smoke	27 (23.3)	13 (22.8)	14 (23.7)
Never smoked	77 (66.4)	40 (70.2)	37 (62.7)
Living arrangement			
Alone	22 (19.0)	10 (17.5)	12 (20.3)
With a partner	66 (56.9)	35 (61.4)	31 (52.5)
With friends or housemates	9 (7.8)	5 (8.8)	4 (6.8)
With parents or relatives	19 (16.4)	7 (12.3)	12 (20.3)
Vegetable intake (serves/day), mean (SD)	2.54 (1.20)	2.58 (1.19)	2.51 (1.22)
Meals eaten with household in last 7 days, mean (SD)	8.94 (5.06)	9.36 (4.6)	8.51 (5.50)
Does main food shopping for household, n (%)	89 (76.7)	47 (82.5)	42 (71.2)
Does main food preparation for household, n (%)	82 (70.7)	41 (71.9)	41 (69.5)
Readiness for change, n (%)			
Took action more than 6 months ago to change eating habits and working hard to maintain this change	10 (8.62)	5 (8.77)	5 (8.47)
Doing something to improve eating habits	33 (28.5)	15 (26.3)	18 (30.5)
Have definite plans to improve eating habits in the next month	27 (23.3)	12 (21.1)	15 (25.4)
Seriously intending to improve eating habits in the next 6 months	40 (34.5)	22 (38.6)	18 (30.5)
Know should improve eating habits, but don’t intend to	6 (5.17)	3 (5.26)	3 (5.08)

1, Rurality based on the Monash Modified Model: MMM2, Regional; MMM4, Large rural towns; MMM5 small rural towns

2, High education (University degree; Higher University degree), medium education (Trade/apprenticeship; Certificate/diploma), low education (no formal qualifications; Year 10 or equivalent; Year 12 or equivalent)

### Digital health equity

Most participants reported using their mobile daily at baseline (intervention: 98%; control; 100%) and 12 weeks (intervention: 95%; control; 100%). At baseline, participants used their devices for, on average, 5.19 (SD 3.0) hours/day in the control group and 5.19 (SD 4.27) hours/day in the intervention group. At 12 weeks this was 4.95 (SD 2.08) hours/day and 5.07 (SD 4.33) hours/day respectively. Most participants reported having access to their own device at baseline (intervention: 100%; control: 100%) and 12 weeks (intervention: 98%; control: 100%). Most participants were extremely confident using computers/mobile devices and getting food-related information via a digital platform at baseline (88% and 79% vs. 84% and 75%, respectively) as well as at 12 weeks (78% and 78% vs. 91% and 76%, respectively).

### Engagement

**Fig. 2:**
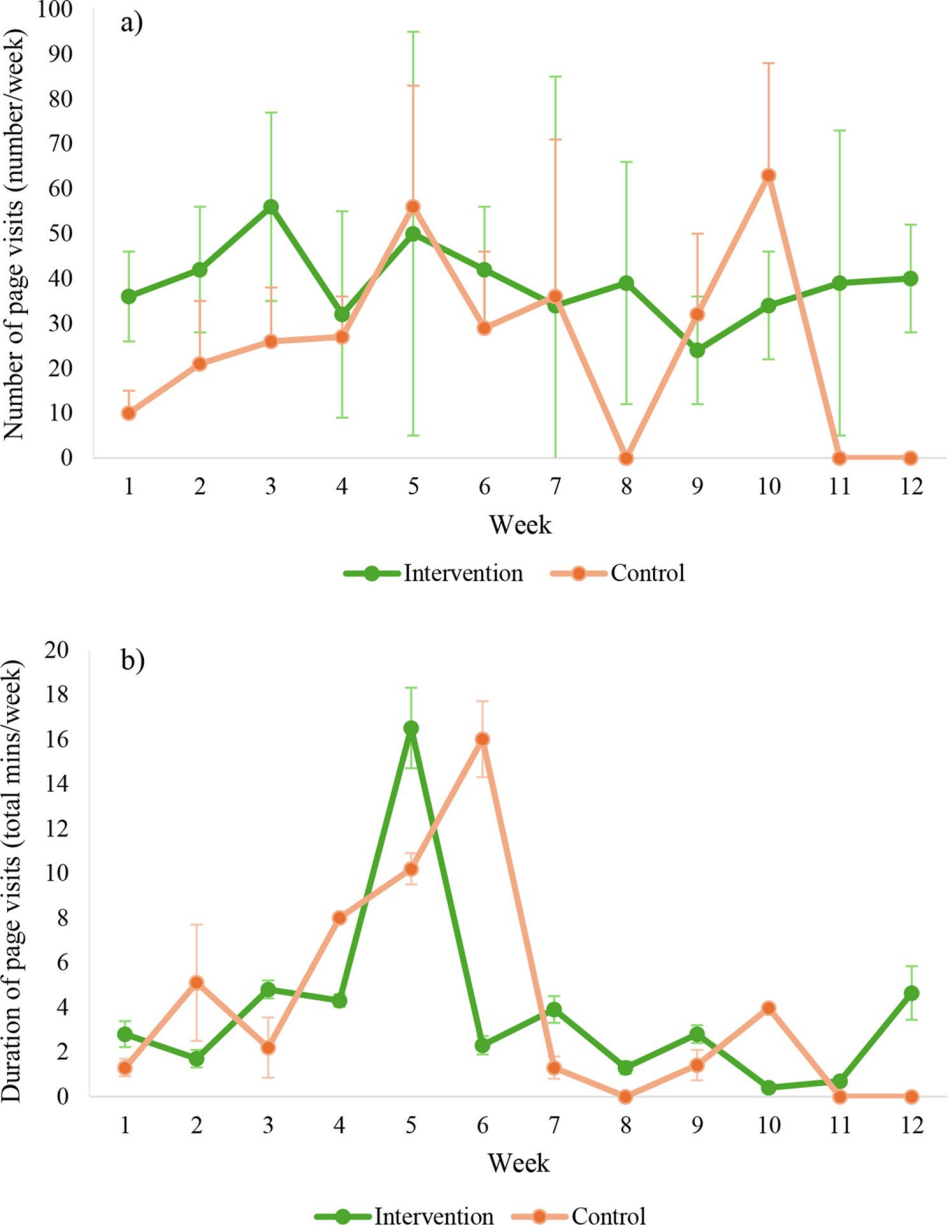
Median (IQR) number of visits to pages (**a**) and median (IQR) duration of page visits (**b**) across the 12 weeks in the control and intervention

The median number of weekly *Veg4Me* page visits was 20 (IQR 3, 54) in the intervention group and 6 (IQR 1, 28) in the control group (Fig. 2). The median duration of page visits was 4.14 (IQR 2.1, 9.0) minutes/week in the intervention group and 2.07 (IQR 0.7, 10.0) minutes/week in the control group. The median number of page visits remained relatively stable in the intervention group, whereas this fluctuated in the control group, with no page visits in weeks 11 and 12 in the control group. The median duration of page visits increased in both groups up until week 5 in the intervention and week 6 in the control, before declining for the remainder of the intervention in both groups. The most frequently visited pages in the intervention group were the food environment map (median 43 [IQR 21, 85] per week), the healthy eating hub (median 41 [IQR 21, 138] per week) and the personalised recipes and recipe library (median 29 [IQR 13, 71] per week). The most frequently visited pages in the control group were the recipe library (median 20 [IQR 9, 59] per week) and list of food environment resources (median 18 [IQR 6, 56] per week).

**Fig. 3:**
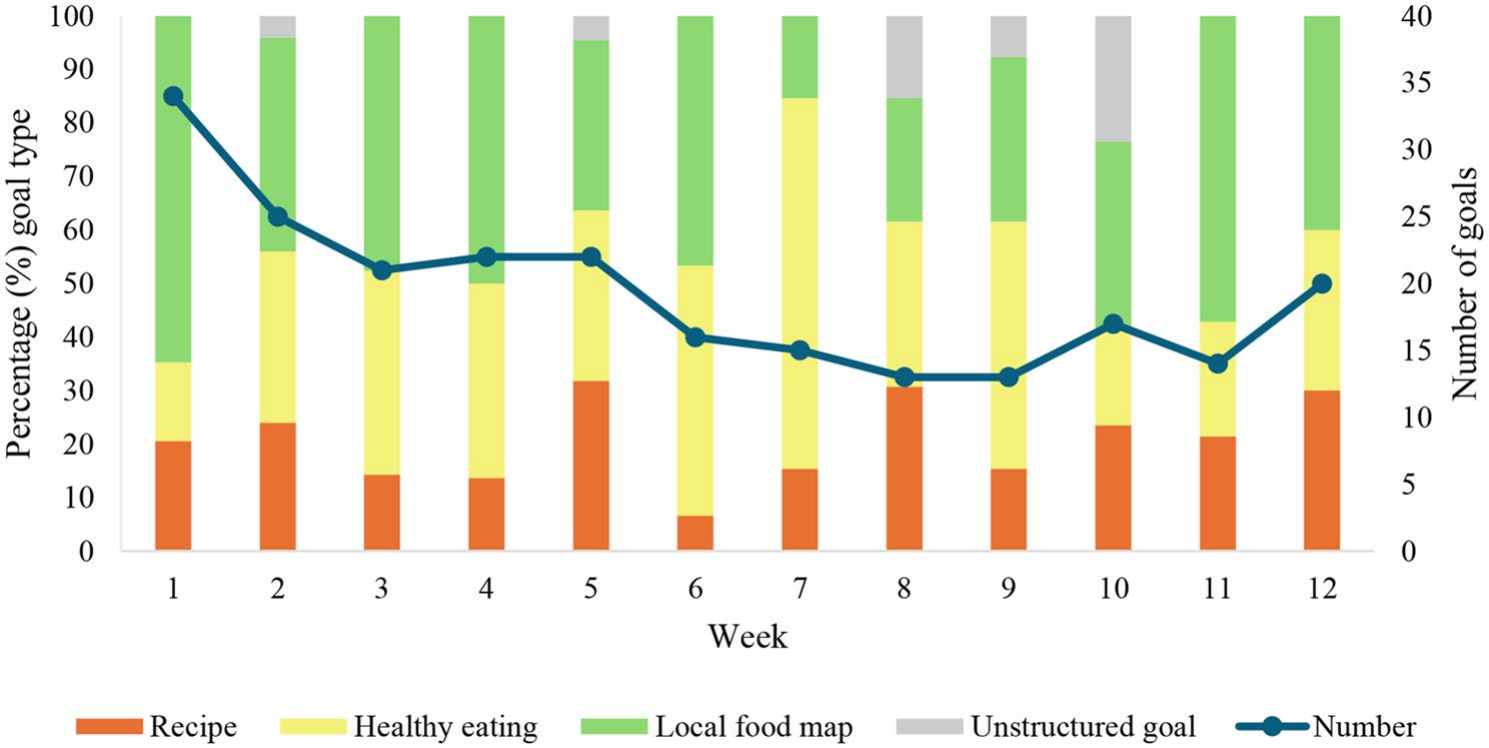
Number of participants who set goals in the 12-week intervention and percentage according to goal type (recipe goal, healthy eating goal, local food environment map goal, unstructured goal)

The percentages of participants who set structured goals for using the recipes, establishing healthy eating habits, accessing the local food environment map and the percentage of participants who set unstructured goals are shown in Fig. 3. The number of intervention participants who set goals (either structured or unstructured) each week ranged from *n* = 34 in week 1 to *n* = 20 in week 12.

The most selected structured goal throughout the 12 weeks was for accessing the local food environment map, followed by establishing healthy eating habits, using the recipes and unstructured goals. For example, in week 1, 65% (*n* = 22) of the participants who set a goal set a structured goal to access a location in their local food environment map, 21% (*n* = 7) set a structured goal to try a recipe and 15% (*n* = 14) set a structured goal to set a healthy eating goal. Similarly, in week 12, 57% (*n* = 8) of participants who set a goal set a structured goal to access a location in their local food environment map, 21% (*n* = 6) set a structured goal to try a recipe and 21% (*n* = 6) set a structured goal to set a healthy eating goal.

Across the 12-week intervention, a total of 9 participants set unstructured goals, most of which were set between weeks 8 and 10 of the intervention. The content of the unstructured goals related to either preparing recipes or healthy eating strategies. For example:

*Use the recipe suggestions at least 3 times this week and encourage all members of household to try a new vegetable” (Participant 134*,* female*,* 34 years)*.

*By Friday I want to use canned beans in at least 2 recipes and take them to work the next day” (Participant 26*,* female*,* 19 years).*

### Acceptability

**Fig. 4:**
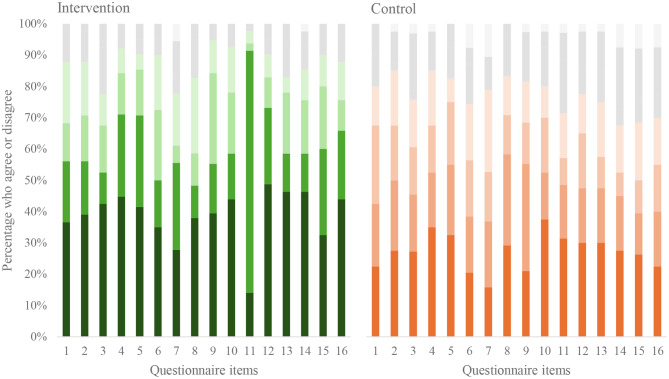
Acceptability of *Veg4Me* using the Post-Study System Usability Questionnaire using a 7-point Likert scale. The darker the colour the more participants agreed with the following questionnaire items: (1) Overall, I am satisfied with how easy it is to use this system; (2) It was simple to use this system; (3) I was able to complete the tasks and scenarios quickly using this system; (4) I felt comfortable using this system; (5) It was easy to learn to use this system; (6) I believe I could become productive quickly using this system; (7) The system gave error messages that clearly told me how to fix problems; (8) Whenever I made a mistake, I could recover easily and quickly; (9) The information provided with this system was clear; (10) It was easy to find the information I needed; 11) The information was effective in helping me complete the tasks and scenarios; 12) The organisation of information on the system screens was clear; 13) The interface of this system was pleasant; 14) I liked using the interface of this system; 15) This system has all the functions and capabilities I expect it to have; 16) Overall, I am satisfied with this system

Compared with the control group, higher proportions of participants in the intervention group reported they were satisfied with *Veg4Me* (76% vs. 52%), that *Veg4Me* was effective in helping them complete tasks and manage scenarios (73% vs. 48%), and that *Veg4Me* has all the functions and capabilities they expected (78% vs. 45%; Fig. 4).

Themes and example quotes for the acceptability of *Veg4Me* are presented in Table 2 for intervention and control participants. Themes identified in intervention participants included “Food literacy”, “Recipes supported healthy eating”, “Local food environment map connected community members” and “Preference for a mobile app”. Participants indicated that *Veg4Me* helped them develop food literacy skills, including food shopping habits, growing their own vegetables and learning about new types and varieties of vegetables and how to cook with them. When asked about the recipe feature, participants indicated that access to recipes supported them to prepare healthy meals. Feedback on the local food environment map indicated that participants liked connecting with their local community. Reasons for not using the local food environment map related to already being aware of what was available in their area, forgetting to try it, no time, or not knowing the resource was available. Reasons for *Veg4Me* not being acceptable to participants in the intervention group largely focused on a preference for a mobile app rather than a web app. Feedback from control participants included the need for personalisation features that were already available in the intervention, such as personalised recipes based on dietary preferences.

**Table 2:** Themes and example quotes for the acceptability of *Veg4Me*

Theme	Example quote
**Intervention group**	
Food literacy	*“From using the app […] I was saving money when shopping and enjoying healthy meals that were not too hard to make :) I even got a small garden going of fresh potato*,* chives*,* herbs. Fresh garlic*,* onion and fruit on my list next!”* (Participant 124, female, 27 years) *“It was interesting to learn different facts about vegetables that I don’t know much about”* (Participant 14, female, 21 years) *“Motivated me to eat different coloured vegetables”* (Participant 18, female, 27 years) *“About using legumes in dishes. I had never done this however now I have multiple times”* (Participant 20, female, 19 years)
Recipes supported healthy eating	*“These recipes helped my support workers plan better food options for my cooking day. We used to just cook cakes and cookies. Now they help me cook veggies and meals.”* (Participant 34, male, 24 years) *“Probably*,* out of all the tools*,* I use the recipes the most. I did think it was a really great variety of different types of recipes and different food from different cultures and that sort of stuff. I definitely was very satisfied with the recipes.”* (Participant 49, female, 22 years)
Local food environment map connected community members	*“Being able to access a local community group was nice for mental health. Finding like-minded people was great for motivation. There’s a positive feeling when connecting with people who were able to help/give advice and also talk through options and give suggestions.”* (Participant 50, female, 28 years)
Preference for a mobile app	*“It is an important and good app but has to be an actual app and not be on a browser”* (Participant 100, male, 30 years) *“It was great content*,* but sometimes I forgot to access it if I didn’t check my emails and get the reminder. Being a web app unless I bookmarked it*,* it was a bit of a process to search the app*,* login and then search recipes”* (Participant 16, female, 27 years)
**Control group**	
Rudimentary tailoring of content	*“It was ok but fairly basic*,* it lacks a bit of fun and ‘wow’. A search function with key terms or ‘tags’ would have been helpful. When I have specified in my profile that I am a vegetarian*,* it is not helpful to have to scroll through lots of chicken recipes to find meat free alternatives.…Just similar little nuances and things you could do in that regard*,* such as the tags for vegetarian options would make it certainly more specialised and personalised for you as a user.”* (Participant 102, female, 33 years)

Most intervention participants (85%) enjoyed receiving 7 new recipes each week, and most indicated that they will continue using these recipes in future (81%; Table 3). 93% of intervention participants liked having access to the recipe library, compared with 76% of control participants. Most intervention participants (95%) agreed the recipes were easy to follow and understand. More intervention participants (83%) agreed that access to recipes gave them confidence to eat a wider variety of vegetables, compared with the control (71%). Similarly, almost twice as many intervention participants (68%) shared the recipes with others, compared with the control (36%).

Among three quarters of intervention participants (76%) who accessed the food environment map, information on farmers markets (68%) and food relief (52%) resources were rated the most useful; information on cooking classes (19%) was the least useful (Table 3). A total of 63% of intervention participants accessed the healthy eating resources, of which the snack and meal ideas were rated the most useful content (96%) and the action tip boxes were the least useful (73%). A total of 78% of intervention participants reported accessing the goal-setting function and almost all participants (90%) reported that the e-newsletters prompted them to access *Veg4Me*.

**Table 3:** Acceptability of *Veg4Me* resources

	Control (*n* = 57) ^6^	Intervention (*n* = 59)^6^
**Recipes,** *** n *(%)**^**1**^		
Overall, I enjoyed receiving 7 new recipes each week	-	35 (85.4)
I liked having access to a recipe library where I could browse other recipes	-	38 (92.7)
Overall, I enjoyed having access to the recipes	32 (76.2)	-
The recipes were easy to access	32 (76.2)	38 (92.7)
The recipes were easy to follow and understand	36 (85.7)	39 (95.1)
The time allocated to prepare/cook recipes was appropriate	35 (83.3)	36 (87.8)
I adapted recipes when I didn’t have all the ingredients listed	29 (73.2)	30 (73.2)
I adapted recipes to suit my taste/dietary preferences	26 (61.9)	32 (78.1)
There was enough variety of recipes with regard to meal types (e.g. breakfast, lunch, dinner, snack, sides)	26 (61.9)	30 (73.2)
There was enough variety of recipes with regard to meal styles (e.g. Asian, Italian)	24 (57.1)	32 (78.1)
I gained more confidence in my cooking skills by following the recipes	21 (50.0)	27 (65.9)
The recipes gave me confidence to eat a wider variety of vegetables	30 (71.4)	34 (82.9)
Trying the recipes encouraged me to find new recipes from other sources	31 (73.8)	31 (75.6)
I have incorporated some of the recipes as regulars in my diet	31 (73.8)	29 (70.7)
I shared the recipes with others (e.g. friends/family)	15 (35.7)	28 (68.3)
I will continue using these recipes in future	31 (73.8)	33 (80.5)
**Local food environment**,** n (%)**^**2**^		
The local food environment map	-	31 (76.0)
The local food environment resources	31 (74.0)	-
Community garden / garden group	12 (38.7)	11 (35.5)
Food relief	17 (54.8)	16 (51.6)
Cooking class	6 (19.4)	6 (19.4)
Community café / lunch	10 (32.3)	17 (54.8)
Farmers market / market	23 (74.2)	21 (67.7)
**Healthy eating hub**,** n (%)**^**2**^		
The healthy eating hub	-	26 (63.4)
General information about vegetables (e.g. health benefits)	-	24 (92.3)
How to choose and store vegetables (including seasonality guide)	-	24 (92.3)
Snack and meal ideas	-	25 (96.2)
Cooking tips with suggested recipes	-	24 (92.3)
Veg fact boxes	-	21 (80.8)
Action tip boxes	-	19 (73.1)
Interactivity of the content (e.g. the ‘click me’ buttons)	-	20 (76.9)
Visual presentation (e.g. colours, images)	-	23 (88.5)
**Goal-setting function**,** n (%)**^**3**^	-	32 (78.1)
**e-newsletters**,** n (%)**^**4**^	-	37 (90.2)

1, 5-point Likert scale responses ranging from “strongly agree” to “strongly disagree” were aggregated, whereby data presented include “strongly agree” and “somewhat agree” with the statement

2, 5-point Likert scale responses ranging from “strongly agree” to “strongly disagree” were aggregated, whereby data presented include “strongly agree” and “somewhat agree” that the resource was useful

3, Data presented are n (%) who reported using the goal-setting function

4, Data presented are n (%) who reported that the e-newsletters prompted them to access the *Veg4Me* web application

5, A total of *n* = 42 participants in the control group and *n* = 41 in the intervention group completed the 12-week intervention

### Efficacy

#### Vegetable intake and dietary habits

The preliminary effects of *Veg4Me* on vegetable intake and dietary habits after 12 weeks are shown in Table 4. Mean vegetable intake was 2.51 (SD 1.2) serves/day at baseline and 2.73 (SD 1.1) serves/day at 12 weeks for the intervention, compared to 2.58 (SD 1.2) serves/day at baseline and 2.66 (SD 1.4) serves/day at 12 weeks for the control group (adjusted mean difference 0.12, 95% CI: -0.41, 0.65; *p* = 0.67). All indicators of confidence to eat healthily over the next year were higher in intervention participants than the control, showing some evidence of efficacy. Similarly, confidence to cook and eat different vegetables was mostly higher among the intervention compared with the control. Compared with the control (57%), more intervention participants (85%) perceived that their vegetable intake had changed in the last 12 weeks (OR 4.07; 95% CI 1.41, 11.8; *p* = 0.01). Some evidence of efficacy was also observed in reported changes in food shopping (*p* = 0.17) and food preparation habits in the last 12 weeks (*p* = 0.09; Table 4). At 12 weeks, more intervention participants were at the ‘action’ stage of readiness for change (49%) compared with control participants (41%), although this difference was not statistically significant (OR 1.32; 95% CI 0.53, 3.28; *p* = 0.55).

### Sensitivity analyses

Effects on vegetable intake and dietary habits at 12-weeks were comparable when a complete case analysis was used (Additional file 2). There was some evidence that participants who were more engaged with the app (*n* = 59) had greater changes in dietary intake and habits at 12 weeks compared with those with low engagement (data not shown). For example, in highly engaged participants, compared with the control, intervention participants reported greater confidence to “shop regularly for healthy nutritious foods” (OR 6.87; 95% CI 1.68, 28.0; *p* = 0.007) and “eat vegetables when I am tired and have to prepare them” (OR 6.77; 95% CI 1.02, 44.9; *p* = 0.048). Similarly, there was some evidence that rural-dwelling participants had greater changes in dietary intake and habits at 12 weeks compared with regional participants (data not shown). For example, compared with the control, rural-dwelling intervention participants reported greater confidence to eat “at least two different vegetables during main meal on most days” (OR 6.47; 95% CI 1.1137.7; *p* = 0.038) and that they had “changed food preparation habits in last 12 weeks” (OR 9.70; 95% CI 1.77, 53.3; *p* = 0.009).

**Table 4:** Effect on vegetable intake and dietary habits at 12-weeks

Dietary indicator	Control (*n* = 57) ^1^		Intervention (*n* = 59)^1^		Difference between groups	
					Odds ratio (95% CI)^2^	*P* value^2^
	**Baseline**	**12 weeks**	**Baseline**	**12 weeks**		
Vegetable intake (serves/day), mean (SD)	2.58 (1.2)	2.66 (1.4)	2.51 (1.2)	2.73 (1.0)	0.12 (-0.41, 0.65)^3^	0.67
Confidence to eat healthy over the next year, n (%)						
Shop regularly for healthy nutritious foods	29 (50.2)	23 (54.8)	26 (44.1)	28 (68.3)	2.36 (0.87, 6.42)	0.09
Prepare/cook healthy nutritious foods	32 (56.1)	24 (57.2)	17 (28.8)	25 (61.0)	1.85 (0.68, 5.00)	0.23
Eat enough vegetables for good health	24 (42.1)	20 (47.6)	14 (23.7)	22 (53.7)	1.95 (0.72, 5.25)	0.19
Confidence to cook vegetables, n (%)						
Pulses (beans and lentils)	19 (33.3)	20 (47.6)	20 (33.9)	22 (53.7)	1.38 (0.52, 3.64)	0.52
Potatoes (not chips)	56 (98.3)	37 (88.1)	51 (86.4)	33 (80.5)	0.87 (0.24, 3.13)	0.84
Fresh green vegetables	53 (93.0)	38 (90.5)	50 (84.8)	38 (92.7)	3.62 (0.42, 31.4)	0.24
Root vegetables	50 (87.7)	34 (81.0)	45 (76.3)	36 (87.8)	5.61 (0.67, 47.0)	0.11
Confidence to eat vegetables, n (%) I can eat.						
Vegetables even when I have to prepare them myself	44 (77.2)	34 (64.3)	40 (67.8)	33 (80.5)	0.97 (0.27, 3.44)	0.96
At least two different vegetables during main meal on most days	38 (66.7)	29 (69.1)	32 (54.2)	29 (70.7)	1.36 (0.50, 3.66)	0.55
Vegetables even on days when I am in a rush	22 (38.6)	24 (57.1)	19 (32.2)	20 (48.8)	0.68 (0.22, 2.09)	0.51
Vegetables when I am tired and have to prepare them	18 (31.6)	18 (42.9)	12 (20.3)	17 (41.5)	1.45 (0.52, 4.04)	0.48
Vegetables when they are mixed with other foods	51 (89.5)	36 (85.7)	45 (76.3)	35 (85.4)	1.29 (0.30, 5.66)	0.73
Vegetables as part of my lunch on most days	22 (38.6)	24 (57.1)	16 (27.1)	17 (41.5)	0.64 (0.23, 1.77)	0.39
Vegetables as a snack at least once a day	10 (17.5)	16 (38.1)	12 (20.3)	16 (39.0)	0.92 (0.33, 2.58)	0.88
Changed vegetable intake in last 12 weeks, n (%)	-	24 (57.1)	-	35 (85.4)	4.07 (1.41, 11.8)	0.010
Changed food shopping habits in last 12 weeks, n (%)	-	23 (54.8)	-	28 (68.3)	1.89 (0.77, 4.64)	0.17
Changed food preparation habits in last 12 weeks, n (%)	-	24 (57.1)	-	31 (75.6)	2.32 (0.89, 6.07)	0.09

1, A total of *n* = 42 participants in the control group and *n* = 41 in the intervention group completed the 12-week intervention

2, Binary logistic models were used to evaluate group differences on outcomes at 12 weeks adjusted for age and baseline levels of the outcome and stratifying factors (region and gender). Missing outcome data was handled using multiple imputation by chained equations with stratification factors and age included as auxiliary variables, with imputation performed separately by study group. No baseline adjustment was available for change in vegetable intake or habits in the last 12 weeks

3, Linear models were used to evaluate group differences in vegetable intake at 12 weeks adjusted for age and baseline levels of the outcome and stratifying factors (region and gender). Missing outcome data was handled using multiple imputation by chained equations with stratification factors and age included as auxiliary variables, with imputation performed separately by study group

## Discussion

Findings from this 12-week pilot RCT demonstrate the feasibility of delivering a co-designed and personalised digital intervention that could be used to increase vegetable intake. Although retention was slightly lower than the target of 80%, targets for recruitment and participation were met. In the personalised intervention group, engagement with the content was higher and more participants reported being satisfied with the intervention, and its features, compared with the non-personalised control. More participants perceived that their vegetable intake had changed in the last 12 weeks compared with the control, however change in reported intake was small. Larger and longer trials of effectiveness are needed to determine whether new accessible and scalable personalisation strategies can help address individual and food environment barriers to healthy eating experienced by young adults living in rural communities.

Retention in digital dietary interventions remains challenging [41, 42]. A recent review of 107 nutrition, physical activity and/or obesity RCTs in young adults (17–35 years) indicated only 65% of studies had adequate retention (≥ 80% retained for ≤ 6-month follow-up or ≥ 70% for > 6-month follow-up) [43], and as little as 14% retention reported at 12 months in a RCT among 18–24 year olds [43]. In the present study, we fell slightly below our 80% target rate, with 72% of participants retained at 12 weeks. Since 90% of participants reported that the e-newsletters prompted them to access *Veg4Me*, this strategy may have been effective in helping to retain participants throughout the 12 weeks as well as maintain engagement with the material on a weekly basis. However, since duration of page visits decreased over time, future research should investigate whether introducing more new material over the intervention would enhance engagement. Although recruitment had to be ceased early, targets for recruitment (≥ 40%) and participation rates (≥ 70%) were met. This may reflect the interest in and demand for rural-centred dietary interventions, which are largely underrepresented and under-funded in research [17, 44]. 

Research suggests that personalised dietary interventions are more effective than generalised interventions [14, 42, 45], which is reflected in this study with higher acceptability of and engagement with the intervention compared with the control, and some evidence of effects on vegetable intake and habits. Even when participants were provided access to the same feature, i.e. the recipe library, participants in the intervention rated this feature higher than in the control group, suggesting that satisfaction with the intervention permeated into their overall satisfaction with the app. Although the duration of page visits was at times higher in the control than the intervention, qualitative data from this study suggest that this may reflect an undesirable need for participants to scroll through the list of recipes or list of food environment resources to find what was relevant for their dietary preferences or location. Therefore, as recommended in a recent systematic review and meta-analysis of personalised mobile technologies for behaviour change [42], system-captured data should be collected and interpreted in conjunction with user-reported data. Moreover, feedback from control participants for recipes included personalision based on being vegetarian, which reinforces that the personalisation features included in the intervention were useful. Additional personalisation features that intervention participants requested included tracking of serves of vegetables across the weeks, which has been shown to effectively increase vegetable intake in another app-based community intervention [46]. 

High acceptability of the intervention may be partly attributable to the central involvement of rural-dwelling young adults in co-designing the features of functions of *Veg4Me*, as well as how they were implemented. To our knowledge, no other web-based dietary interventions have included both food literacy resources and a feature to support local food environments [16]. Most interventions focus on developing food literacy skills in young adults within university settings [25, 47] which often are not applicable to young adults in rural settings, or incentive-based interventions which encourage healthy food choices in rural retail settings [48] but do not build better connected and more resilient food systems. While all *Veg4Me* features were well accepted by young adult participants, the personalised recipes were the most highly rated and among the most frequently visited across the 12-week intervention. This confirms previous research highlighting the importance of building capacity for behaviour change by supporting cooking and food skills in young adults [49], and personalising the intervention to dietary preferences [45]. Interestingly, the most widely used goals related to accessing the food environment map, suggesting that people may not need to set recipe goals as often since the personalised recipes were made available on a weekly basis, and thus used the goal-setting feature to support their use of the local food environment map. The most useful resource in the map was farmers’ markets, which may reflect the desire of rural young adults to support local producers [50]. These findings highlight the unique value of an intervention that simultaneously addresses capacity, opportunity and motivation to change behaviour by building confidence to grow, store and prepare vegetables while also addressing barriers to accessing affordable and fresh produce [23]. 

Efficacy outcomes for vegetable intake and dietary habits observed in this study are comparable with previous research [51]. Although between-group differences in vegetable intake were modest (+ 0.12 serves/day), and most differences in outcomes were not statistically significant, our findings align with estimates of mean change in vegetable consumption from a recent review of effective interventions [51]. Moreover, this feasibility trial consistently demonstrated numerically greater confidence to eat healthily and to cook vegetables across all outcomes. The vegetable of the week feature was intended to support young adults to establish self-efficacy to cook with different vegetable types, which appears to have been successful, with more intervention participants reporting confidence to cook with pulses, potatoes, fresh green vegetables and root vegetables than in the control. As most participants were at the ‘contemplation’ stage of behaviour change at baseline and most intervention participants were at the ‘action’ stage at follow-up, further research should explore whether being part of the intervention aided participants to progress through the transtheoretical model of change, i.e. pre-contemplation, contemplation, preparation, action and maintenance [33]. Given the low intake of vegetables in young adults [5], and limited culinary use of pulses in many Western cultures [52], these findings are encouraging. Similar findings have been observed in a 6-week RCT to improve the nutrition, food literacy, and cooking skills of low-socioeconomic Australian adults, where the food literacy program improved cooking confidence, food preparation behaviours, nutrition knowledge and vegetable consumption significantly [53]. Since dietary interventions in regional, rural and remote communities are limited [16, 17], the promising findings from intervention, particularly in rural-dwelling communities, highlights the opportunity for web-based dietary interventions to help reduce health inequities.

Findings from this study have implications for future research. A larger and longer trial of effectiveness and implementation potential is needed to determine whether this co-designed and personalised intervention significantly improves and sustains changes in vegetable intake and whether it can be implemented at scale across more rural communities. A broader range of secondary dietary outcomes should also be considered, assessed using validated tools, to understand impacts on overall diet quality. Given feedback from participants largely focused on the delivery mode of the intervention, future research should consider the development of a mobile app. Given this is a public health intervention that is not focused on weight loss, future research may also consider broadening the eligibility to include breastfeeding mothers, who represented a large proportion of interested, but screened out, individuals. Further, qualitative data suggested that the intervention impacted on the diets of family members and households, therefore there may be a need to target intervention material according to household needs, and an opportunity to measure changes in vegetable intake of other family/ household members. Action was taken to make the intervention accessible, however, it targeted individuals with access to an internet-connected device; therefore, future interventions would need to target priority populations to optimise digital health equity, such as remote and low educated individuals.

The present study had several strengths. A major strength was that the intervention used in this study was conceptualised and co-designed from iterative workshops that enabled the local community and key stakeholders to explore, conceptualise and refine ideas for the intervention features and functions. The web-based delivery of the intervention was a strength as it enabled the intervention to be accessible to a wide geographic region, whilst providing links to place-based initiatives for participants to use to feel connected to their local community. The integration of behaviour change techniques and features to address barriers to vegetable intake via both food literacy skills and the food environment enabled participants to change their behaviours via a combination of capacity, opportunity and motivation. Lastly, the combination of built-in tracking data from the web platform, the use of quantitative surveys and the inclusion of interviews enabled an in-depth understanding of the feasibility of the trial.

This study had several limitations. Restricting recruitment to two particular geographic regions limits generalisability to the wider rural Australian population. However, it was appropriate to first test the feasibility in the geographic region where the intervention was co-designed, prior to testing in a larger and more representative sample. The sample was female-skewed, which may limit generalisability of findings to other genders. However, most participants were responsible for household food shopping and food preparation, thereby providing a pathway to improve vegetable intake by other household or family members, including males. Future research should measure vegetable intake by other household members to determine total intervention effects. Whilst the highest proportion of participants in this trial resided in regional areas, there was good representation of participants from both large and small rural towns. While the target recruitment sample size was not reached, due to limits on the funding period, we exceeded our target rate for converting eligible individuals into consented participants. This bodes well for a longer effectiveness trial. Although the target of 80% retention was not met, targets for recruitment and participation were met. The present retention rate was comparable to adequate retention rates for studies with > 6 months follow up (> 70%), which requires testing in a trial of effectiveness. The collection of self-reported data lacked detailed and objective insights into dietary behaviours and findings may be confounded by social desirability bias. The functions and features of the app were designed within the scope of a feasibility trial, which limited the ability to meet the digital technology expectations of all participants. Lastly, effects should be interpreted with caution since this feasibility study was not powered to detect effects of the intervention.

## Conclusion

A 12-week co-designed personalised digital intervention (*Veg4Me*) was feasible to deliver, with promising findings for engagement, acceptability and efficacy. Whilst outcomes need to be confirmed in a larger trial, these findings provide insights into personalised digital strategies to help address individual and food environment barriers to healthy eating experienced by young adults living in rural communities.

## Electronic supplementary material

Below is the link to the electronic supplementary material.

Supplementary Material 1

Supplementary Material 2

## Data Availability

The datasets generated and/or analysed during the current study are not publicly available but may be made available by the first author on reasonable request and upon approval by the Deakin University Human Research Ethics Committee.

## Abbreviations

COM-B: Capability, Opportunity and Motivation Behaviour Change model
CONSORT: Consolidated Standards of Reporting Trials
TIDieR: Template for Intervention Description and Replication
COREQ: COnsolidated criteria for REporting Qualitative research
MM: Monash Model
NAASS: Non-adoption, Abandonment, Scale-up, Spread, and Sustainability
PSSUQ: Post-Study System Usability Questionnaire
SMART: Specific, Measurable, Achievable, Relevant, and Time-Bound
