# Supplementary material for: Feasibility of a co-designed and personalised intervention to improve vegetable intake in rural-dwelling young adults
Source: Int J Behav Nutr Phys Act. 2025 Jul 14;22:97. doi: 10.1186/s12966-025-01796-7 (PMC12257653; doi:10.1186/s12966-025-01796-7)
Supplement: Supplementary file 1 — Supplementary Material 1 [file 12966_2025_1796_MOESM1_ESM.docx]

**Supplemental Table 1.** TIDieR (Template for Intervention Description and Replication) checklist

| **Item number** | **Item** | **Where located** |
| --- | --- | --- |
|  | **BRIEF NAME** |  |
| **1.** | Provide the name or a phrase that describes the intervention. | 6 |
|  | **WHY** |  |
| **2.** | Describe any rationale, theory, or goal of the elements essential to the intervention. | 6–7 |
|  | **WHAT** |  |
| **3.** | Materials: Describe any physical or informational materials used in the intervention, including those provided to participants or used in intervention delivery or in training of intervention providers. Provide information on where the materials can be accessed (e.g. online appendix, URL). | 9–11 |
| **4.** | Procedures: Describe each of the procedures, activities, and/or processes used in the intervention, including any enabling or support activities. | 9–11 |
|  | **WHO PROVIDED** |  |
| **5.** | For each category of intervention provider (e.g. psychologist, nursing assistant), describe their expertise, background and any specific training given. | N/A |
|  | **HOW** |  |
| **6.** | Describe the modes of delivery (e.g. face-to-face or by some other mechanism, such as internet or telephone) of the intervention and whether it was provided individually or in a group. | 9–11 |
|  | **WHERE** |  |
| **7.** | Describe the type(s) of location(s) where the intervention occurred, including any necessary infrastructure or relevant features. | 9–11 |
|  | **WHEN and HOW MUCH** |  |
| **8.** | Describe the number of times the intervention was delivered and over what period of time including the number of sessions, their schedule, and their duration, intensity or dose. | 9 |
|  | **TAILORING** |  |
| **9.** | If the intervention was planned to be personalised, titrated or adapted, then describe what, why, when, and how. | 9–11 |
|  | **MODIFICATIONS** |  |
| **10.** | If the intervention was modified during the course of the study, describe the changes (what, why, when, and how). | N/A |
|  | **HOW WELL** |  |
| **11.** | Planned: If intervention adherence or fidelity was assessed, describe how and by whom, and if any strategies were used to maintain or improve fidelity, describe them. | 12–13 |
| **12.** | Actual: If intervention adherence or fidelity was assessed, describe the extent to which the intervention was delivered as planned. | 16 |

**Supplementary Table 2.** CONSORT checklist of information to include when reporting a pilot trial

| **Section/topic and item No** | **Standard checklist item** | **Extension for pilot trials** | **Page No where item is reported** |
| --- | --- | --- | --- |
| Title and abstract | | | |
| 1a | Identification as a randomised trial in the title | Identification as a pilot or feasibility randomised trial in the title | 1 |
| 1b | Structured summary of trial design, methods, results, and conclusions (for specific guidance see CONSORT for abstracts) | Structured summary of pilot trial design, methods, results, and conclusions (for specific guidance see CONSORT abstract extension for pilot trials) | 4–5 |
| Introduction | | | |
| Background and objectives: | | | |
| 2a | Scientific background and explanation of rationale | Scientific background and explanation of rationale for future definitive trial, and reasons for randomised pilot trial | 6–7 |
| 2b | Specific objectives or hypotheses | Specific objectives or research questions for pilot trial | 7 |
| Methods | | | |
| Trial design: | | | |
| 3a | Description of trial design (such as parallel, factorial) including allocation ratio | Description of pilot trial design (such as parallel, factorial) including allocation ratio | 7 |
| 3b | Important changes to methods after trial commencement (such as eligibility criteria), with reasons | Important changes to methods after pilot trial commencement (such as eligibility criteria), with reasons | N/A |
| Participants: | | | |
| 4a | Eligibility criteria for participants |  | 7–8 |
| 4b | Settings and locations where the data were collected |  | 7–8 |
| 4c |  | How participants were identified and consented | 7–9 |
| Interventions: | | | |
| 5 | The interventions for each group with sufficient details to allow replication, including how and when they were actually administered |  |  |
| Outcomes: | | | |
| 6a | Completely defined prespecified primary and secondary outcome measures, including how and when they were assessed | Completely defined prespecified assessments or measurements to address each pilot trial objective specified in 2b, including how and when they were assessed | 9–11 |
| 6b | Any changes to trial outcomes after the trial commenced, with reasons | Any changes to pilot trial assessments or measurements after the pilot trial commenced, with reasons | 16 |
| 6c |  | If applicable, prespecified criteria used to judge whether, or how, to proceed with future definitive trial | 12 |
| Sample size: | | | |
| 7a | How sample size was determined | Rationale for numbers in the pilot trial | 14 |
| 7b | When applicable, explanation of any interim analyses and stopping guidelines |  | N/A |
| Randomisation: | | | |
| Sequence generation: | | | |
| 8a | Method used to generate the random allocation sequence |  | 9 |
| 8b | Type of randomisation; details of any restriction (such as blocking and block size) | Type of randomisation(s); details of any restriction (such as blocking and block size) | 9 |
| Allocation concealment mechanism: | | | |
| 9 | Mechanism used to implement the random allocation sequence (such as sequentially numbered containers), describing any steps taken to conceal the sequence until interventions were assigned |  | 9 |
| Implementation: | | | |
| 10 | Who generated the random allocation sequence, enrolled participants, and assigned participants to interventions |  | 9 |
| Blinding: | | | |
| 11a | If done, who was blinded after assignment to interventions (eg, participants, care providers, those assessing outcomes) and how |  | 9 |
| 11b | If relevant, description of the similarity of interventions |  | N/A |
| Analytical methods: | | | |
| 12a | Statistical methods used to compare groups for primary and secondary outcomes | Methods used to address each pilot trial objective whether qualitative or quantitative | 14–15 |
| 12b | Methods for additional analyses, such as subgroup analyses and adjusted analyses | Not applicable | 15 |
| Results | | | |
| Participant flow (a diagram is strongly recommended): | | | |
| 13a | For each group, the numbers of participants who were randomly assigned, received intended treatment, and were analysed for the primary outcome | For each group, the numbers of participants who were approached and/or assessed for eligibility, randomly assigned, received intended treatment, and were assessed for each objective | Figure 1 |
| 13b | For each group, losses and exclusions after randomisation, together with reasons |  | 16 |
| Recruitment: | | | |
| 14a | Dates defining the periods of recruitment and follow-up |  | 4–5, 16 |
| 14b | Why the trial ended or was stopped | Why the pilot trial ended or was stopped | 16 |
| Baseline data: | | | |
| 15 | A table showing baseline demographic and clinical characteristics for each group |  | 18 |
| Numbers analysed: | | | |
| 16 | For each group, number of participants (denominator) included in each analysis and whether the analysis was by original assigned groups | For each objective, number of participants (denominator) included in each analysis. If relevant, these numbers should be by randomised group | Figure 1 |
| Outcomes and estimation: | | | |
| 17a | For each primary and secondary outcome, results for each group, and the estimated effect size and its precision (such as 95% confidence interval) | For each objective, results including expressions of uncertainty (such as 95% confidence interval) for any estimates. If relevant, these results should be by randomised group | 15–33 |
| 17b | For binary outcomes, presentation of both absolute and relative effect sizes is recommended | Not applicable | N/A |
| Ancillary analyses: | | | |
| 18 | Results of any other analyses performed, including subgroup analyses and adjusted analyses, distinguishing prespecified from exploratory | Results of any other analyses performed that could be used to inform the future definitive trial | 16–34 |
| Harms: | | | |
| 19 | All important harms or unintended effects in each group (for specific guidance see CONSORT for harms) |  | 16 |
| 19a |  | If relevant, other important unintended consequences | N/A |
| Discussion | | | |
| Limitations: | | | |
| 20 | Trial limitations, addressing sources of potential bias, imprecision, and, if relevant, multiplicity of analyses | Pilot trial limitations, addressing sources of potential bias and remaining uncertainty about feasibility | 36–37 |
| Generalisability: | | | |
| 21 | Generalisability (external validity, applicability) of the trial findings | Generalisability (applicability) of pilot trial methods and findings to future definitive trial and other studies | 36–37 |
| Interpretation: | | | |
| 22 | Interpretation consistent with results, balancing benefits and harms, and considering other relevant evidence | Interpretation consistent with pilot trial objectives and findings, balancing potential benefits and harms, and considering other relevant evidence | 33–37 |
| 22a |  | Implications for progression from pilot to future definitive trial, including any proposed amendments |  |
| Other information | | | |
| Registration: | | | |
| 23 | Registration number and name of trial registry | Registration number for pilot trial and name of trial registry | Universal Trial Number U1111-1284-9027; please see ACTRN 12623000179639 |
| Protocol: | | | |
| 24 | Where the full trial protocol can be accessed, if available | Where the pilot trial protocol can be accessed, if available | doi: 10.1136/bmjopen-2023-078001 |
| Funding: | | | |
| 25 | Sources of funding and other support (such as supply of drugs), role of funders |  | 4 |
| 26 |  | Ethical approval or approval by research review committee, confirmed with reference number | 36 |
