# Supplementary material for: Feasibility of a co-designed and personalised intervention to improve vegetable intake in rural-dwelling young adults
Source: Int J Behav Nutr Phys Act. 2025 Jul 14;22:97. doi: 10.1186/s12966-025-01796-7 (PMC12257653; doi:10.1186/s12966-025-01796-7)
Supplement: Supplementary file 2 — Supplementary Material 2 [file 12966_2025_1796_MOESM2_ESM.docx]

**Supplementary Table 3.** Preliminary effect on vegetable intake and dietary habits at 12-weeks using a complete case analysis approach

| **Dietary indicator** | **Control (n=57) ^1^** | | **Intervention (n=59)^1^** | | **Difference between groups** | |
| --- | --- | --- | --- | --- | --- | --- |
|  |  |  |  |  | **Odds ratio**  **(95% CI)^2^** | **P value^2^** |
|  | **Baseline** | **12 weeks** | **Baseline** | **12 weeks** |  |  |
| Vegetable intake (serves/day), mean (SD) | 2.58 (1.2) | 2.66 (1.4) | 2.51 (1.2) | 2.73 (1.0) | 0.11 (-0.42, 0.64)^3^ | 0.68 |
| Does food shopping in household, n (%) | 47 (82.5) | 32 (56.1) | 42 (71.2) | 41 (69.5) | 1.66 (0.76, 3.65) | 0.21 |
| Does food preparation in household, n (%) | 41 (71.9) | 57 (100) | 41 (69.5) | 59 (100) | - | - |
| Confidence to eat healthy over the next year, n (%) |  |  |  |  |  |  |
| Shop regularly for healthy nutritious foods | 29 (50.2) | 23 (54.8) | 26 (44.1) | 28 (68.3) | 2.25 (0.84, 6.02) | 0.11 |
| Prepare/cook healthy nutritious foods | 32 (56.1) | 24 (57.2) | 17 (28.8) | 25 (61.0) | 1.71 (0.63, 4.61) | 0.29 |
| Eat enough vegetables for good health | 24 (42.1) | 20 (47.6) | 14 (23.7) | 22 (53.7) | 1.77 (0.68, 4.65) | 0.25 |
| Confidence to cook foods, n (%) |  |  |  |  |  |  |
| Pulses (beans and lentils) | 19 (33.3) | 20 (47.6) | 20 (33.9) | 22 (53.7) | 1.40 (0.54, 3.63) | 0.48 |
| Potatoes (not chips) | 56 (98.3) | 37 (88.1) | 51 (86.4) | 33 (80.5) | 0.83 (0.22, 3.19) | 0.78 |
| Fresh green vegetables | 53 (93.0) | 38 (90.5) | 50 (84.8) | 38 (92.7) | 4.94 (0.60, 40.8) | 0.14 |
| Root vegetables | 50 (87.7) | 34 (81.0) | 45 (76.3) | 36 (87.8) | 7.64 (0.88, 66.5) | 0.07 |
| Confidence to eat vegetables, n (%) I can eat.. |  |  |  |  |  |  |
| Vegetables even when I have to prepare them myself | 44 (77.2) | 34 (64.3) | 40 (67.8) | 33 (80.5) | 0.89 (0.25, 3.17) | 0.86 |
| At least two different vegetables during main meal on most days | 38 (66.7) | 29 (69.1) | 32 (54.2) | 29 (70.7) | 1.23 (0.46, 3.29) | 0.68 |
| Vegetables even on days when I am in a rush | 22 (38.6) | 24 (57.1) | 19 (32.2) | 20 (48.8) | 0.63 (0.22, 1.78) | 0.38 |
| Vegetables when I am tired and have to prepare them | 18 (31.6) | 18 (42.9) | 12 (20.3) | 17 (41.5) | 1.35 (0.47, 3.87) | 0.58 |
| Vegetables when they are mixed with other foods | 51 (89.5) | 36 (85.7) | 45 (76.3) | 35 (85.4) | 1.10 (0.25, 4.72) | 0.90 |
| Vegetables as part of my lunch on most days | 22 (38.6) | 24 (57.1) | 16 (27.1) | 17 (41.5) | 0.54 (0.20, 1.47) | 0.23 |
| Vegetables as a snack at least once a day | 10 (17.5) | 16 (38.1) | 12 (20.3) | 16 (39.0) | 0.87 (0.32, 2.36) | 0.78 |
| Changed vegetable intake in last 12 weeks, n (%) | - | 24 (57.1) | - | 35 (85.4) | 4.30 (1.45, 12.7) | 0.008 |
| Changed food shopping habits in last 12 weeks, n (%) | - | 23 (54.8) | - | 28 (68.3) | 1.79 (0.72, 4.46) | 0.21 |
| Changed food preparation habits in last 12 weeks, n (%) | - | 24 (57.1) | - | 31 (75.6) | 2.09 (0.79, 5.51) | 0.14 |

## 1, A total of n=42 participants in the control group and n=41 in the intervention group completed the 12-week intervention

2, Binary logistic models were used to evaluate group differences on outcomes at 12 weeks adjusted for baseline levels of the outcome and stratifying factors (age, region and gender). Missing outcome data was handled using multiple imputation by chained equations with stratification factors and age included as auxiliary variables, with imputation performed separately by study group. No baseline adjustment was available for change in vegetable intake or habits in the last 12 weeks.

3, Linear models were used to evaluate group differences in vegetable intake at 12 weeks adjusted for baseline levels of the outcome and stratifying factors (age, region and gender). Missing outcome data was handled using multiple imputation by chained equations with stratification factors and age included as auxiliary variables, with imputation performed separately by study group
